# Some Alkaloidal Specialities

**Published:** 1908-04-18

**Authors:** 


### NEW APPLIANCES AND THINGS MEDICAL.

**We shall be glad to receive, at our Office, 28 & 29 Southampton Street, Strand, London, W.C., from the manufacturers, specimens of all new preparations and appliances which may be brought out from time to time.]**

### SOME ALKALOIDAL SPECIALITIES.

(The Abbott Alkaloidal Company, Chicago. London Agents: Basing House, Basinghall Street, E.C.)

We have received from this firm an assortment of their newest alkaloidal preparations, many of which are deserving of the attention of general practitioners. All the products manufactured by this well-known company enjoy a well-deserved reputation for excellence in America. They have been thoroughly tested and found efficient, and are well worth a trial by practitioners here. *Abbott's Saline Laxative* is a most efficient aperient and one of the best of the manufactured Seidlitz-salt preparations which we have so far seen. It consists of chemically-pure sulphate of magnesium in effervescent combination, and is a safe, pleasant, non-griping laxative. Its usefulness in constipation and in some varieties of diarrhœa cannot be over-estimated. The *Nuclein* preparations made by the same firm are put up in half-ounce bottles as solutions or in tablets. The value of nuclein is perhaps underestimated by the profession here; certainly it is by no means so largely or so systematically used as in America. The preparation of the Abbott Company is suitable alike for hypodermic or internal administration, and has been used with success in typhoid, septicæmia, and debilitated conditions generally. Nuclein is a powerful tonic, and is certainly deserving of more attention from the general practitioner than it has hitherto received. *Calcalith* is the catalogue name for one of the company's preparations recommended as "an efficient remedy for all the manifestations of the uric acid diathesis." It is a mixture of the carbonates of calcium and lithium with small doses of colchicine. It appears to be a useful and pleasant-tasting preparation, and in two cases in which we have seen it used it acted with excellent results. The dose of colchicine contained in each dose of calcalith is exceedingly small, and in some cases its use will have to be supplemented by a saline laxative. *Calcidin* is the company's trade name for a preparation of iodised lime (calx iodata), which has been employed with marked benefit in cases of catarrh, influenza, and bronchitis. It is put up in the form of tablets which are easily soluble. Another important preparation that well merits trial is the *Abbott Intestinal Antiseptic*, which is a tablet containing a mixture of the sulphocarbolates of calcium, sodium, and zinc. In several cases of diarrhœa its usefulness was plainly demonstrated, and it is manifestly a most useful preparation.

One of the specialities of the Abbott Alkaloidal Company is the preparation known as the *Hyoscine-Cactine-Morphine Anæsthetic*, which we hope to report on at a future date, when we have had an opportunity of testing its merits. The preparation is at the present time much used in America, but appears to be wholly unknown here, where its place is taken by the far less efficient and more dangerous scopolamine compound. With all these preparations the company issues booklets containing full directions and much useful information regarding treatment. Each preparation is well "written up," and the practitioner who desires to give a fair and unprejudiced trial to these American novelties cannot do better than write for samples of the preparations and accompanying literature and investigate the merits of the many "products" stocked at Basing House. All the preparations are tastefully put up and are permanent.

